# Determinants of long acting and permanent contraceptive methods utilization among married women of reproductive age groups in western Ethiopia: a cross-sectional study

**DOI:** 10.11604/pamj.2015.21.246.5835

**Published:** 2015-08-06

**Authors:** Alemu Sufa Melka, Tesfalidet Tekelab, Desalegn Wirtu

**Affiliations:** 1 College of Medical and Health Sciences, Wollega University, Nekemte, Oromia, Ethiopia

**Keywords:** Contraception, long acting, permanent methods, western Ethiopia

## Abstract

**Introduction:**

In Ethiopia information on the level of utilization of the long term and permanent contraceptive methods and associated factorsis lacking. The aim of this study was to understand the determinant factors of long acting and permanent contraceptive methods use among married women of reproductive age in Western Ethiopia.

**Methods:**

A community based cross- sectional study design was employed. Multi stage sampling was used to select 1003 study participants. Data was collected from April 10 to April 25,2014 using a pre- tested structured questionnaire. The data were entered using Epi-info version 3.5.1 and exported to SPSS version 20 for analysis. Multivariate logistic regression analysis was done to identify predictors of long acting and permanent contraceptive methods at 95% CL.

**Results:**

Use of long acting and permanent contraceptive methods in this study was found to be 20%. Survey results showed a significant positive association between utilization of long acting and permanent contraceptive methods and women's education (AOR = 1.72, 95%CI = 1.02 - 3.05), women's occupation (AOR = 2.01, 95% CI = 1.11 -3.58), number of live children (AOR = 2.42, 95% CI: 1.46- 4.02), joint fertility related decision (AOR = 6.11, 95% CI: 2.29- 16.30), having radio/TV (AOR = 2.31, 95% CI: 1.40 - 3.80), and discussion with health care provider about long acting and permanent contraceptive methods (AOR = 13.72, 95% CI: 8.37 - 22.47).

**Conclusion:**

Efforts need to be aimed at women empowerment, health education, and encouraging open discussion of family planning by couples

## Introduction

Couples who want safe and effective protection against pregnancy would benefit from access to more contraceptive choices, including long-acting and permanent methods (LAPMs). LAPMs include: IUDs, implants, Tubal ligation, and vasectomy. These are by far the most effective (>99%) methods of contraception available and are very safe and convenient. The long term nature of these forms of contraception does not require daily motivation on the part of users and thus have higher continuation and effectiveness rates. Couples also require fewer visits to health providers, thus saving time, effort and money, and the patient load at health care facilities is lessened [1].

This means that they are cost effective for programs over time. At a system level, the use of LAPMs can result in substantial cost savings for governments, and contribute directly to reaching national and international health goals. Other indirect advantages are the reduction of high maternal mortality and morbidity, as well as elective abortions. According to a study done in different nations across the globe, more than 300,000 abortions per year in Vietnam, more than 100,000 in Ukraine, and 80,000 in Turkey could be averted by the use of LAPMs [2].

Despite these advantages, LAPMs remain a relatively small, and sometimes missing, component of many national reproductive health and family planning programs. Strong family planning programs offer a full range of contraceptive methods, but in many places, LAPMs are the least available, the least used, and possibly the least understood methods by client [1, 3].

For example, evidence from sub-Saharan Africa suggests there is a large discrepancy between the proportion of women who want to stop having children and the proportion using LAPM. This implies large unmet need for LAPMs [4].

The Ethiopia Federal Ministry of Health (FMoH) has considered the important role of LAPMs and aims to increase the availability of these methods to 20% of all family planning clients [5]. Use of family planning in Ethiopia has traditionally focused on short-acting methods such as injectable and birth control pills. LAPMs accounted for only 4% of users in 2011 in Ethiopia. High discontinuation rates are associated with the short-acting hormonal methods, such as injectable, that are predominantly chosen by contraceptive users in sub-Saharan Africa. However, increasing contraceptive method mix has been shown to reduce discontinuation rates [5, 6].

Most research to date in Ethiopia has concentrated on examining factors that influence all modern contraception methods. The present study emphasizes on the determinant factors of LAPM methods utilization in Western Ethiopia with the aim of providing policy makers and program managers findings that contribute to the improvement of service provision with regard to LAPMs.

## Methods

### Study design, setting and participants

A community-based cross- sectional study was employed from April 10 to April 25, 2014 among married women of reproductive age in Nekemte town, Oromia Region, West Ethiopia. Nekemte town is a capital of East Wollega Zone (Province) located at 321 km from Addis Ababa. The total population of the town is estimated to be 75,219 of which 38,385(51%) are females [7]. All ever married women aged 15-49 years and lived in the study area at least for 6 months were included in the study. Women who were critically ill, mental incapable to provide informed consent and infecund were excluded from the study.

### Sample size and sampling procedures

The sample size was determined using a formula for estimation of single population proportion with the assumption of 95% confidence interval, 3% margin of error, and 12.3% prevalence of LAPM use [8], and design effect of 2. To compensate for the non-response rate, 10% of the determined sample was added up on the calculated sample size and the final sample size was 1012. A multi-stage sampling technique was employed for the selection of the sampling units. Three sub-cities were selected from the six in the town of Nekemte, followed by the random selection of four zones from each the sub-cities. The calculated sample sizes for these zones were proportionally allocated based on the number of married women living in each of them. One house was randomly selected as the initial household in each zone, and the final households with married women were selected using systematic random sampling from the existing sampling frame of households. Finally, eligible married women of reproductive age in the selected households were asked to participate in the study. When two or more married women were in a household, only one of them was randomly asked to participate, to avoid intra-class correlation.

### Data collection procedures

A pre-tested structured questionnaire was adapted from different literature[5, 8–10]. The English language questionnaire was translated into the regional language of Afan Oromo, and then translated back to English by other people who are proficient in both languages to maintain the consistency of the questionnaires. Five high-school completed females administered the structured questionnaire, after a 4-day training session that included information about the objective and relevance of the study, confidentiality of information, participants’ rights, informed consent, interview techniques, and practical demonstration of the interview. Four degree-prepared colleagues supervised the data collection procedures. Supervision involved reviewing all questionnaires at the end of every day, followed by morning meetings with the data collectors to discuss on any problems encountered during data collection.

### Data processing and analysis

Data was cleaned and entered into a computer using Epi-info Window version 3.5.1 statistical program. Then the data was exported to SPSS Windows version 20.0 for analysis. The descriptive analysis including proportions, percentages, frequency distribution and measures of central tendency was done. Initially, bivariate analysis was performed between dependent variable and each of the independent variables, one at a time. Their odds ratios (OR) at 95% confidence intervals (CI) and p-values were obtained, to identify important candidate variables for multivariate analysis. All variables found to be significant at bivariate level (at p-value

### Ethical Considerations

Ethical clearance and permission was obtained from Wollega University Institutional Review Board. Permission was secured from all sub cities of Nekemte town through a formal letter. Written Informed consent were obtained from each respondent before interviewing. The written informed consent was also includes study participants less than 18 years since they were married and minor mature and the consent procedure was approved by ethics committee of Wollega University. Confidentiality of individual client information was ensured by using unique identifiers for study participants and limiting access to the principal investigator and research assistants of study information by storing the completed questionnaires and all documents with participant information in a lockable cabinet.

## Results

### Socio-demographic characteristics

A total of 1003married women of reproductive age completed the questionnaire making a response rate of 99%. About half of the participants (51.6%) were in the age group of 25-34 years with mean age of 28.3 years (SD+ 6.1 years). Three quarters of the respondents (74.8%) were from the Oromo ethnic group. About half of the respondents (48.4%) were protestant in religion. Two hundred ninety one (27.9%) of the respondents had completed secondary education while 11.3% could not read and write. More than half of the respondents (52.9%) were housewives. Information about the husbands of the participants was also collected: 29.4% of them had attended college or higher,5.9% could not read and write, and 42.7% of them were daily laborers. Their mean monthly income was ETB 1510.6. Out of the total married women 72.1% had radio/TV (Table 1).

**Table 1:** Socio demographic characteristics of Married women in Nekemte town, Nekemte, Ethiopia, April, 2014

Variables (1003)	Number (%)
Age category	
15-24	281 (28.0)
25-34	514 (51.2)
35-44	198(19.7)
>44	10 (1.0)
Ethnicity	
Oromo	750(74.8)
Amhara	200(19.9)
Tigre	36(3.6)
Others*	17(1.7)
Religion	
Protestant	485(48.4)
Orthodox	423(42.2)
Catholic	17(1.7)
Muslim	76(7.6)
Others**	2(0.2)
Educational status of the respondent	
Can't read and write	113(11.3)
Can read and write	71(7.1)
G1-4	142(14.2)
G5-8	206(20.5)
Secondary	280(27.9)
College and above	191(19.0)
Educational status of the husband	
Can't read and write	59(5.9)
Can read and write	48(4.8)
G1-4	81(8.1)
G5-8	249(24.8)
Secondary	271(27.0)
College and above	295(29.4)
Occupational status of the respondents	
Governmental Employee	157 (15.7)
Daily laborer	157(15.7)
Housewife	531(52.9)
Merchant	99(9.9)
Student	53 (5.3)
Others	6 (0.6)
Occupational status of the Husband	
Governmental Employee	359(35.8)
Daily laborer	428(42.7)
Merchant	153(15.3)
Student	19(1.9)
Others****	44(4.4)
Income (ETB)	
<600	221(22.0)
600-1000	275(27.4)
1001-1500	135(13.5)
1501-2000	179(17.8)
>2000	193(19.2)
Mean	1533 ETB
Have radio/TV	
Yes	723 (73.1)
No	280(27.9)

* Other = Gurage, Shinasha,

** other = Wakefeta, Jehovah's Witness,

*** other = petty maker, house maid,

**** other = Driver, carpenter 1$ = 20ETB

### Fertility and reproduction related characteristics

The majority of the participants (95.8%) experienced pregnancy at least once during the study period and the mean number of living child was 2.39. Three hundred sixty three (37.8%) of the study subjects had less than or equal to two living children. More than half (53.2%) of the study participant expressed a desire for more children in the future. Out of those who desired to have children62.9% desired to have two and less than two children. From those who desired to have children in the future 70.4% of the respondents need more children because they have few children. Half of the partners of the respondents (50.3%) desired to have children in the future. And, the majority of the respondent (84.8%) decided on fertility issue jointly with their husbands. The main reason for not using LAPMs, for the majority (49.3%) of the married women was fear of side effect associated with the methods (Table 2).

**Table 2:** Fertility desire and reproductive history of married women in Nekemte town, Nekemte, Ethiopia, March to April, 2014

Variables	Number (%)
Have you ever pregnant (1003)	
Yes	961(95.8)
No	42(4.2)
Number of children alive (961)	
= < 2	598(62.2)
>2	363(37.8)
Future fertility desire (1003)	
Yes	534(53.2)
No	398(39.7)
I don't know	71(7.1)
Number of desired child (385)	
1-2	242(62.9)
>2	143(37.1)
Reason for future child desire (534)	
Have few children	376(70.4)
Need of son	102(19.1)
Death of child	20(3.8)
No response	48(9.0)
Other*	6(1.1)
Partner fertility desire (1003)	
Yes	505(50.3)
No	370(36.9)
Don't know	128(12.8)
Decision on fertility (1003)	
Wife	36(3.6)
Husband	116(11.6)
Jointly	851(84.8)
Reason for nonuse of LAPMs (768)	
fear of side effect	379(49.3)
Lack of awareness of the LAPM	203(26.4)
Rumors they are not good	199(25.9)
Influence of other Important people	61(7.9)
Not my preferred method	338(44.0)
To have more children	225(29.3)
Husband disapproval	86(11.2)
Religion prohibition	26(3.4)
Fear of infertility	260(33.9)

* Other = Partner wants more children, others influence

### Awareness, ever use and current use of LAPMs of family planning

**Figure 1:**
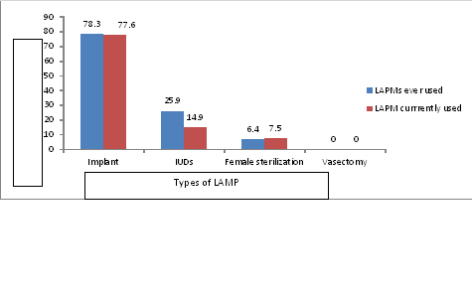
Types of LAMPs ever used and currently used by married women Nekemte town Nekemte, Ethiopia, March to April, 2014

From the total study participants 85.9% of them heard of at least one method of LAPM and 79.2% of them heard about LAPMs from health workers. The most common type of LAPM known was implant (86.1%), and although the majority of participants (88.5%) identified hospital as a place to get LAPMs, only 43.2% of the respondents actually discussed LAPMs with service providers. The ever use and current use prevalence of long acting and permanent contraceptive methods use were 23.4% and 20% respectively (Table 3). Majority of women ever and currently used implants (78.3% and 77.6 respectively) (Figure 1).

**Table 3:** LAPMs use & need of married women in Nekemte town, Nekemte, Ethiopia, March to April, 2014

Variables	Number (%)
Ever heard of LAPM methods(1003)	
Yes	862(85.9)
No	141(14.1)
Source of information on LMPM (862)	
Health worker	683(79.2)
Radio	629(72.9)
TV	632(73.3)
Friends	177(20.5)
Other*	15(1.7)
Type of LAMPS methods ever Heard (862)	
IUCD	771(89.4)
Implant	742(86.1)
Female sterilization	293(34.9)
Vasectomy	137(15.9)
Perceived Source of LAMPs (862)	
Hospital	763(88.5)
Health center	374(43.4)
Health post	203(23.5)
FGA	400(46.4)
Private clinic	251(29.1)
Ever discussion of LAPMs with health worker (1002)	
Yes	433 (43.2)
No	569 (56.8)
Ever use of LAMPs (1003)	
Yes	235(23.4)
No	768(76.6)
Current use of LAPMs(1003)	
Yes	201(20)
No	802(80)

### Factors Associated with current utilization of LAPMs

A multivariate analysis involving all associated variables was performed to identify independent predictors of utilization of long acting and permanent contraceptive methods. Women's education, women's occupation, number of live children, joint decision on fertility, having radio/TV and discussion with health care provider about LAPMs showed significant association with use of long acting and permanent contraceptive methods. Those respondents who had secondary school education and above were more likely to utilize LAPMs compared to those who had primary school education and below AOR = 1.72,95%CI = 1.02 - (3.05’> 95%CI = 1.02 - 3.05). Women who were government employed were two times more likely to use LAPMs than others occupation (AOR = 2.01, 95% CI = 1.11 - 3.58). Women who had more than two children were two times more likely to utilize LAPMs compared to those who had less than or equal to two children (AOR = 2.42,95% CI: 1.46- 4.02). Women's who had joint discussion with their husband on fertility issue were six times more likely to practice LAMPs compared to those who had no joint discussion (AOR = 6.11,95% CI: 2.29- 16.30). Respondents who had radio/TV were more likely to use LAPMs compared to those who had no radio (AOR = 2.31,95% CI: 1.40 - 3.80). Women-s who had discussion with health professionals about LAPMs were fourteen times more likely to use LAPMs than those who had no discussion (AOR = 13.72,95% CI: 8.37 - 22.47) (Table 4).

**Table 4:** A multivariate logistic regression on predictors of use of long acting and permanent contraceptive methods in Nekemte town, Nekemte, Ethiopia, April, 2014

Characteristics	Using LAPMs		*Crude OR*	*Adjusted OR*
	Yes (%)	No (%)	*OR(CI)*	*OR(CI)*
Age categoryin years				
15-24	42(14.9%)	239(85.1%)	1	1
25-34	100(19.5%)	414(80.5%)	1.38(0.927 – 2.038)	0.79(0.45 – 1.38)
35-44	57(28.8%)	141(71.2%)	2.30(1.47 – 3.61)	0.71(0.35 – 1.44)
>44	2(20.0%)	8(80.0%)	1.42(0.29 - 6.93)	0.25(0.03 – 1.66)
Education of respondents				
Below and Primary	66(87.6%)	466(12.4%)	1	1
Secondary and above	135(28.7%)	336(71.3%)	2.84(2.05 – 3.93)	1.72(1.02 – 3.05)*
Education of Husband				
Below and Primary	58(13.3%)	379(86.7%)	1	1
Secondary and above	143(25.3%)	423(74.7%)	2.21(1.58 – 3.09)	0.16(0.67 – 1.97)
Occupation of respondents				
Government Employed	59(37.6%)	98(62.4%)	2.99(2.06 – 4.32)	2.0(1.11 – 3.58)*
Others	142(16.8%)	704(83.2%)	1	1
Husband Occupation				
Government Employed	88 (24.5%)	271(75.5%)	1.53(1.11 – 2.09)	0.85(0.52 – 1.38)
Others	113(17.5%)	531(82.5%)	1	1
Monthly income				
<600	38 (17.2%)	183(82.8%)	1	1
600-1000	35(12.7%)	240(87.3%)	0.70(0.43 – 1.16)	0.83(0.44 – 1.57)
1001-1500	30 (22.2%)	105(77.8%)	1.38(0.81 – 2.35)	0.90(0.45 – 1.81)
1501-2000	46(25.7%)	133(74.3%)	1.67(1.03 – 2.70)	0.59(0.28 – 1.22)
>2000	52(26.9%)	141(73.1%)	1.78(1.11 – 2.85)	0.44(0.20 – 0.95)
Number of live children				
< = 2	85(14.2%)	513(85.8%)	1	1
>2	106(29.2%)	257 (70.8%)	2.49(1.80 – 3.44)	2.42(1.46 – 4.02)*
Respondent Wants more child				
Yes	76(14.2%)	458(85.8%)	1	1
No	125(26.7%)	344 (73.3%)	2.19(1.59– 3.01)	1.65(0.67 – 4.11)
Husband desire more child				
Yes	76(15.0%)	429(85.0%)	1	1
No	125(25.1%)	373 (74.9%)	1.89(1.38 – 2.60)	1.01(0.42 – 2.43)
Joint decision on fertility with partner				
Joint decision	196(23%)	655(77)	8.80(3.56 – 21.76)	6.11(2.29 – 16.30)*
Others	5(3.3)	147(96.7)	1	1
Have radio/TV				
Yes	170(23.5%)	429(85.0%)	2.47(1.64 – 3.72)	2.31(1.40 – 3.80)*
No	125(25.1%)	373 (74.9%)	1	1
Discussion on LAPMs with health worker				
Yes	178(41.1%)	553(76.5%)	16.60(10.49 – 26.27)	13.72(8.37 – 22.47)*
No	31(11.1%)	249 (88.9%)	1	1

* Key: Statistically significant (p-value <0.05) 1-: Reference category

## Discussion

In the current study knowledge of at least one form of LAPMs were high (85.9%). The findings were much higher than previous studies in Ethiopia [5, 8, 9]. The reason for observed difference could be due to information provision by health extension workers, different NGO's and presence of different Medias in the area.

The result of this study showed that 20% of respondents were currently using long acting and permanent contraceptive methods. The finding of this study was similar to other study conducted in Debre Markos Ethiopia, which was 19.5%. The FMOH of Ethiopia has considered the important role of long-acting and permanent methods and aims to provide 20% of all family planning clients with these long-acting and permanent methods [10]. The finding was higher compared to other studies done in Ethiopia[8, 9]. This may be due to increased awareness of communities about long acting and permanent contraceptive methods and promotion about long acting and permanent contraceptive methods by governmental and non-governmental organization found in the town.

Majority of women in this study used implants (77.6%) followed by IUCD (14.9%) and tubal ligation (7.5%). There were no respondents who used vasectomy. This finding is inconsistent with studies done in Turkey and Kenya. This could be lack of awareness on permanent contraceptive methods (tubal ligation and Vasectomy) in this study area [11, 12]. In multivariable analysis educational attainment was important predictors of long acting and permanent contraceptive methods. In this study, women who had secondary education and higher were 1.7 times more likely practiced currently LAPMs than those with lower and primary education. This agrees with studies done in Uganda, Ghana and Zimbabwe [13–15]. The possible explanation could be that better educated women would likely have access for information on modern contraceptive methods and their increased knowledge on modern contraceptives. It is thought that increased educational attainment particularly secondary education influence service use and female decision-making power on reproductive health issues particularly family planning.

This study showed that government employed women were twice as likely to use long acting and permanent contraceptive compared with others. This study is inconsistent with the study done in Gobatown [9]. The finding was agrees with study done in Dembia District and Kelala Town [16, 17]. This may be due to government employed women have more access to information and services of reproductive health.

In this study participants with greater than two children were twice more likely to be currently using LAPMs compared to those with less than and equal to two living children. The finding was in agreement with findings from Ethiopia and Tanzania [8, 18]. Some myths and misbeliefs towards LAPM, such as these methods causing infertility, could be one of the reasons for non-use [19]. As the number of children increase fear of infertility related to those methods would decreased and people tends to use LAPMs.

This study indicated that availability of radio/TV had significant contribution in promoting current utilization of LAPMs. Women who have radio/TV were more likely to use LAPMs compared to those who did not have radio/TV. Exposure to information on television and radio can increase knowledge and awareness of new ideas, social changes, and opportunities and can affect an individual's perceptions and behavior, including those about health [5]. Thus national and local media should have airtime to encourage utilization of LAMPs and remove misconception on LAPMs.

In this study, discussion with health workers about LAPMs was another important predictor of current utilization of LAPMs of contraception. Those women who had discussed these methods with health workers were 14 times more likely to practice LAPMs compared to their counterparts. Similar previous studies also found that discussion between health workers and users was important predictors of LAPMs utilization [9]. Such discussions include counseling and information-sharing about family planning including LAPMs are important in correcting myths and misconceptions related to LAPMs use, and creating awareness of the various LAPMs options that are available for clients to make their preferred choices of FP methods.

Couples who made joint fertility decisions were 6 times more likely to use LAPMs than women who did not involve their husband in fertility decisions. Studies done in Ethiopia and Zambia were consistent with our study [9, 20]. Women participation in household decisions including those related to fertility as well as spousal communication on family planning have been indicated in several studies to be associated with increased likelihood of modern contraceptive use by women [13, 21, 22]. In Ethiopia 32% of those who were involved in decision making about their own health care used a contraceptive method compared to 20% of those who were not involved in decision making regarding their own health care [23]. The finding highlights the importance of couple encouragement and male involvement in reproductive health issues including fertility and contraception. Therefore family planning counseling and information should encourage joint discussion of fertility issues among couples.

The limitation of this study was cross-sectional nature of the data that could obscure the causal effect relationships of different factors. The strength of this study was large sample size used which would more represent the target population.

## Conclusion

The finding of this study showed that 20% of married women currently using long acting and permanent contraceptive methods. Factors which were significantly associated with utilization of long acting and permanent contraceptive methods were women's educational level, women's occupation, number of live children, fertility related decision, having radio/TV and discussion with health care provider about long acting and permanent contraceptive methods. Efforts need to be aimed at women empowerment, health education, and strategies to encourage more open discussion of family planning by couples.

## References

[1] US Agency for International DevelopmentLong-Acting and Permanent Methods: Addressing Unmet Need for Family Planning in Africa.

[2] Bradley SEK, Croft TN, Rutstein SO (2011). The impact of contraceptive failure on unintended births and induced abortions: Estimates and Strategies for Reduction, in DHS analytical studies 2011.

[3] Nancy Y, Stella B (2011). A qualitative study of the use of long-acting and permanent methods of contraception (LA/PMS) in Cambodia.

[4] Family Health International (2008). Addressing unmet need for family planning in Africa: Long-acting and permanent methods. Family Health Research.

[5] (2012). Central Statistical Agency Ethiopia and ORC Macro Ethiopia Demographic and Health Survey (EDHS) 2011.

[6] Scott A, Glasier A (2006). Evidence based contraceptive choices. Best Practice and Research Clinical Obstetrics &Gynaecology..

[7] FDRE The 2007 population and Housing Census of Ethiopia, Results for Oromia Region Part V. Statistical Report on Population and Household size of Kebeles.

[8] Alemayhu M, Belachew T, Tilhaun T (2012). Factors associated with utilization of long acting and permanent contraceptive methods among married women of reproductive age in Mekelle town, Tigray region, North Ethiopia. BMC Pregnancy and Childbirth..

[9] Takele A, Degu G, Yitayal MD (2012). Demand for long acting and permanent methods of contraceptives and factors for non-use among married women of Goba Town, Bale Zone, South East Ethiopia. BMC Reproductive Health..

[10] Bulto GA, Zewdie TA, Beyen TK (2014). Demand for long acting and permanent contraceptive methods and associated factors among married women of reproductive age group in DebreMarkos Town, North West Ethiopia. BMC Women’s Health..

[11] Kahraman K, Goc G, Taskin S, Haznedar P, Karagozlu S (2012). Factors influencing the contraceptive method choice: a university hospital experience. J Turkish-German Gynecol Assoc..

[12] Alaii J, Nanda G, Njeru A (2012). Fears, Misconceptions, and Side Effects of Modern Contraception in Kenya: Opportunities for Social and Behavior Change Communication.

[13] Khan S, Bradley S, Fishel J, Mishra V (2008). Unmet Need and the Demand for Family Planning in Uganda: Further Analysis of the Uganda Demographic and Health Surveys, 1995-2006.

[14] Adanu RM, Seffah JD, Hill AG, Darko R, Duda RB, Anarfi JK (2009). Contraceptive use by women in Accra, Ghana: Results from the 2003 Accra women's health survey. Afr J Reprod Health..

[15] Mturi A, Joshua K (2011). Falling fertility and increase in use of contraception in Zimbabwe. Afr J Reprod Health..

[16] Kebede Y (2006). Contraceptive prevalence in Dembia District, Northwest Ethiopia. Ethiop J Health Dev..

[17] Gizat M, Alemayehu L, Besufekad A (2014). Assessments of Patterns and Determinants of Contraceptive Use among Females of Reproductive Age in Kelala Town, Northern Ethiopia. The Experiment..

[18] Lwelamira J, Mnyamagola G, Msaki MM (2012). Knowledge, Attitude and Practice (KAP) towards Modern Contraceptives among married women of reproductive Age in Mpwapwa District, Central Tanzania. Cur Res J Soc Sci..

[19] Adeleye OA, Akoria OA, Shuaib ZO, Ogholoh OD (2010). Barriers and knowledge of benefits regarding family planning methods among women attending antenatal clinics in a southern Nigerian community. Asian J Med Sci..

[20] Mutombo N, Bakibinga P (2014). The effect of joint contraceptive decisions on the use of Injectables, Long-Acting and Permanent Methods (ILAPMs) among married female (15-49) contraceptive users in Zambia: a cross-sectional Study. BMC reproductive health journal..

[21] Amaha H, Enkusilase F (2006). Influence of women's autonomy on couple's contraception use in Jimma town, Ethiopia. Ethiopian J Health Dev..

[22] Stephenson R, Baschieri A, Clements S, Hennink M, Madise N (2007). Contextual influences on modern contraceptive use in sub-Saharan Africa. Am J Publ Health.

[23] Tadesse M, Teklie H, Yazew G, Gebreselassie T Women's Empowerment as a Determinant of Contraceptive use in Ethiopia Further Analysis of the 2011 Ethiopia Demographic and Health Survey.

